# The Effects of Instructions on Dual-Task Walking and Cognitive Task Performance in People with Parkinson's Disease

**DOI:** 10.1155/2012/671261

**Published:** 2012-12-29

**Authors:** Valerie E. Kelly, Alexis J. Eusterbrock, Anne Shumway-Cook

**Affiliations:** Department of Rehabilitation Medicine, University of Washington, 1959 NE Pacific Street, P.O. Box 356490, Seattle, WA 98125, USA

## Abstract

Gait impairments are prevalent among people with Parkinson's disease (PD). Instructions to focus on walking can improve walking in PD, but the use of such a cognitive strategy may be limited under dual-task walking conditions, when walking is performed simultaneously with concurrent cognitive or motor tasks. This study examined how dual-task performance of walking and a concurrent cognitive task was affected by instructions in people with PD compared to healthy young and older individuals. Dual-task walking and cognitive task performance was characterized under two sets of instructions as follows: (1) focus on walking and (2) focus on the cognitive task. People with PD and healthy adults walked faster when instructed to focus on walking. However, when focused on walking, people with PD and young adults demonstrated declines in the cognitive task. This suggests that dual-task performance is flexible and can be modified by instructions in people with PD, but walking improvements may come at a cost to cognitive task performance. The ability to modify dual-task performance in response to instructions or other task and environmental factors is critical to mobility in daily life. Future research should continue to examine factors that influence dual-task performance among people with PD.

## 1. Introduction

Gait impairments are common in people with Parkinson's disease (PD) and are associated with increased disability, reduced quality of life, and increased risk for falls [1–4]. Gait impairments in PD are exacerbated when walking is performed simultaneously with another task [5–9], which is referred to as dual-task walking. People with PD report that walking while performing another task is one of the greatest challenges of daily mobility [10]. They also describe using concentration to monitor and correct walking [10], consistent with James Parkinson's original observation that, “Walking becomes a task that cannot be performed without considerable attention” [11].

Gait impairments in PD are an important target of therapeutic interventions because of their prevalence and consequences. The use of cognitive processes to consciously attend to and modify gait parameters is a key strategy for gait rehabilitation in PD. For example, people with PD can increase gait speed and stride length when instructed to focus on taking longer strides [12–14]. Such cognitive strategies improve walking under single-task conditions, but the evidence for transfer to dual-task walking conditions is mixed [13, 15]. The ability to improve dual-task walking using cognitive strategies requires that people with PD focus on walking while also directing cognitive resources or processes to the performance of a concurrent cognitive or motor task. Research examining the ability to modify dual-task performance among people with PD is limited.

The mechanisms responsible for interference during dual-task walking in PD are not well understood, but a number of potentially overlapping mechanisms have been proposed [16]. Reduced movement automaticity as a result of basal ganglia dysfunction may increase reliance on cognitive resources or processes to control walking, thereby limiting the use of cognition to perform concurrent cognitive or motor tasks. Inappropriate prioritization, referred to as “posture second” prioritization, has also been proposed as a mechanism contributing to dual-task walking deficits in people with PD [17]. An inability to direct cognitive resources to walking under dual-task conditions may contribute to unsafe or inappropriate prioritization of concurrent tasks over walking, potentially contributing to an increased risk for falls during dual-task walking. While dual-task walking deficits are well established in PD, the degree to which people with PD can modify dual-task walking is not clear. A better understanding of the factors that influence the ability to modify dual-task performance is necessary to develop more effective interventions for walking under dual- and multi-task conditions that are common in daily life.

The purpose of this research was to study the effects of instructions on dual-task performance of walking and a concurrent cognitive task in people with PD compared to healthy individuals. We hypothesized that people with PD would retain the ability to modify dual-task performance in response to instructions to focus on walking or the cognitive task, suggesting that task prioritization is dynamic, rather than fixed. However, because it has been suggested that people with PD use cognitive resources in the control of walking, we anticipated that the ability to modify dual-task performance would differ from healthy individuals in two ways. First, we expected that among people with PD, improvements in walking performance in response to instructions to focus on walking would be associated with declines in concurrent cognitive task performance. Second, we anticipated that an increased use of cognitive resources to control walking would limit the relative magnitude of dual-task performance changes in people with PD compared to healthy young and older adults. Understanding factors that influence dual-task performance in people with PD can inform the development of interventions to improve dual-task walking in this population.

## 2. Methods

### 2.1. Participants

We recruited healthy young adults (HYA) from the university community and healthy older adults (HOA) from local exercise classes and the community. We recruited participants with PD from the community and a state registry program. Participants with PD were included if they had a clinical diagnosis of PD and were excluded if they had a history of surgery for PD. Exclusion criteria for all participants were a diagnosis of dementia or any other neurologic or orthopedic condition that affected the ability to walk 200 feet without assistance, cognition, or the ability to complete the protocol. A phone screen was used to determine eligibility based on inclusion and exclusion criteria, and all eligible participants were invited to participate in the study. We determined a target sample size of 10–19 using a priori power calculations from a range of published differences in dual-task gait speed between healthy individuals and people with PD. Written informed consent was obtained prior to data collection in accordance with approved institutional review board procedures at the University of Washington Human Subjects Division (057021A01). 

### 2.2. Data Collection and Analysis

Participants attended a single testing session at a university-based motion analysis laboratory. Baseline characteristics of age, number of medical conditions, medications, severity of motor symptoms in PD, and cognitive function were recorded. People with PD were tested in the medication-on condition, with the assessment of walking beginning within 1-2 hours after taking antiparkinson medications. Motor symptoms of PD were characterized using the Movement Disorders Society Unified Parkinson's Disease Rating Scale (MDS-UPDRS) Part III, Motor Examination and Hoehn and Yahr staging. Cognitive function for HOA and people with PD was assessed using the Montreal Cognitive Assessment (MoCA) [18].

For the cognitive task, participants completed an auditory Stroop test [19, 20] consisting of the words “high” or “low” said in a high or a low pitch. Participant were instructed to “respond as quickly and as accurately as possible” by verbally identifying the pitch. Each trial was 3 seconds in length, with a variable 0-1 second delay before stimulus presentation. A wireless headset and microphone system (Plantronics, Inc., Santa Cruz, USA; Jabra Corporation, Nashua, USA) was integrated with custom hardware and software for data collection. All groups performed three seated training blocks of 20 stimuli per block to minimize learning effects. For the remainder of testing, blocks were 20 stimuli for HYA and 8–12 stimuli for HOA and people with PD. Block length was consistent for a given participant, but was adjusted between participants to ensure capture of a similar number of strides per condition while minimizing fatigue. Stimuli were pseudorandomized to ensure equal representation of each of the four possible stimuli. Single-task blocks of the cognitive task were performed in sitting at the beginning and the end of the testing session. Two blocks were performed in each dual-task condition. Primary outcome measures for the cognitive task were response latency and response accuracy. These outcome measures were chosen because both were emphasized in the instructions and because changes in cognitive task performance could result from changes in either or both measures. Response latency was measured as the time from stimulus onset to response onset. Response accuracy was the number of correct responses divided by the total number of stimuli, expressed as a percentage.

For the walking task, participants were instructed to “walk as quickly as safely possible” in a taped pathway (8.8 m length; 60 cm width) on level ground. Participants walked with their arms crossed to eliminate the use of the arms for balance and to allow adequate motion capture for whole-body modeling. A Qualisys Motion Capture system (Qualisys, Gothenburg, Sweden) recorded the position of markers placed bilaterally on the feet (calcaneus, lateral malleolus, 3rd metatarsal-phalangeal joint), legs (tibial tuberosity, lateral knee joint, superior patella, mid-thigh, greater trochanter), pelvis (anterior superior iliac spine, iliac crest), and trunk (sternum, thorax, acromion). Whole-body center of mass was calculated as the weighted sum of an 8-segment model (bilateral feet, shanks, and thighs, pelvis, and combined head, arms, and trunk segment). Participants walked with their arms crossed for all trials. Two blocks (60 seconds for HYA, 24–36 seconds for HOA and people with PD) were recorded for each condition as participants walked back and forth across the walkway, with only straight ahead walking recorded and analyzed. Walking was characterized using gait speed as the primary outcome measure. 

In addition, secondary gait parameters were measured to further characterize spatiotemporal changes and stability. Spatiotemporal measures were stride length, cadence, and step width. Stability measures included step width variability (coefficient of variation) to assess the consistency spatial parameters, stride time variability (coefficient of variation) to assess the consistency of temporal parameters, and center of mass frontal plane inclination angle to assess biomechanical stability. Step width variability has been proposed to reflect postural control while walking [21], while stride time variability is thought to reflect the rhythmicity of walking [22]. Inclination angle has been proposed as a biomechanical measure of postural control while walking [23]. The inclination angle was calculated at each heel strike as the angle between the line connecting the center of mass and the lateral ankle marker and the vertical line through the whole body center of mass in the frontal plane [23, 24]. The inclination angle provides a measure of the position of the center of mass relative to the base of support (approximated by the ankle joint).

In order to examine the effects of instructed focus on dual-task performance, two different instructions were provided during dual-task walking. Instructions to focus on one task versus another have been shown to impact dual-task walking in healthy young [25] and older adults [26]. In the cognitive task focus condition (DT_cog_), instructions were “focus on the cognitive task, and perform it as quickly and as accurately as when you were sitting.” For the walking focus condition (DT_walk_), instructions were “focus on walking, and walk as quickly as when you were only walking.” The order of conditions was randomized.

The effect of instructions on dual-task walking and cognitive task performance was assessed by comparing (1) absolute measures of performance and (2) the dual-task effect (DTE) in the DT_cog_ and DT_walk_ conditions. The DTE is a relative measure of dual-task compared to single-task performance and was calculated for each of the primary outcome measures as the difference between single-task and dual-task performance, normalized to single task performance and expressed as a percentage. All DTEs were operationally defined such that a negative value represents a dual-task cost or decrement and a positive value represents a dual-task benefit [20]. DTEs for response latency and response accuracy were summed to create a composite cognitive task DTE for each individual. The composite cognitive task DTE was used to assess overall cognitive task performance, as it helped to account for potential within-task trade-offs or varying patterns of decline in latency versus accuracy.

The effect of instructions was also assessed using the modified attention allocation index. The attention allocation index has been used previously to measure the ability to allocate attention in response to instructed focus [19, 27]. We used a modified version of the attention allocation index (mAAI) to evaluate the capacity to modify performance based on instructed focus [20]. The mAAI for each variable was calculated as the difference in DTE in the DT_walk_ condition and the DTE in the DT_cog_ condition. The mAAI was operationally defined such that positive values indicate a performance shift toward the instructed task (i.e., instructed task improves), and negative values indicate a shift away from the instructed task (i.e., instructed task declines). 

### 2.3. Statistical Analysis

Descriptive analysis was performed for all variables (SPSS Statistics version 17.0, Chicago, USA). Potential baseline group differences in age and cognitive function (HOA and people with PD only) were assessed using *t*-tests. Single-task walking and cognitive task performance were compared across groups using repeated measures analysis of variance (ANOVA) with one between-subject factor (GROUP: HYA, HOA, PD). The effects of instructions on absolute and relative measures of dual-task performance were examined with a repeated measures ANOVAs using two instructions (INSTRUCTIONS: DT_walk_, DT_cog_) and one between-subject factor (GROUP: HYA, HOA, PD). The mAAI for walking and the cognitive task were compared among groups using ANOVA with one between-subject factor (GROUP). The level of significance for all tests was set at *α* = .05. Effect size for all ANOVAs was reported using partial eta squared (*η*_*p*_^2^), with a small effect defined as 0.0099, a medium effect as .0588, and a large effect as .1379 [28]. When ANOVAs were statistically significant, post hoc comparisons were performed using the Scheffé test. With Bonferroni correction, the level of significance for post hoc comparisons was set at *α* = .017. Effect size for all post hoc comparisons was reported using Cohen's *d*, with a small effect defined as .2, a medium effect as .5, and a large effect as .8 [29]. 

## 3. Results

### 3.1. Baseline Characteristics

Table 1 shows the individual characteristics and group summary for the 15 participants with idiopathic PD and the group summaries for the 15 HYA and 15 HOA. The mean age of the HYA was 26.4 (SD = 4.3) years, and the HOA and people with PD were similar in age (HOA: 69.2 [7.1]; PD: 72.2 [6.2]; *P* = .23). There were more men in the PD group than in the HYA or HOA groups. Participants with PD had mild to moderate disease severity as indicated by MDS-UPDRS scores and Hoehn and Yahr staging. There was a trend toward lower MoCA scores among people with PD compared to HOA (*P* = .07). On average, young adults had 0.2 (0.6) medical diagnoses and were taking 0.7 (1.1) medications, while older adults had 1.9 (1.7) medical diagnoses and were taking 2.3 (2.6) medications, consistent with healthy samples and low disease burden. People with PD had 2.7 medical diagnoses (including PD) and were taking 1.7 (0.9) antiparkinson medications and 2.4 (2.2) medications unrelated to PD on average. The average levodopa equivalent dose [30] was 265 (238) mg.

### 3.2. Single-Task and Dual-Task Performance: Absolute Measures

Table 2 shows the results for absolute measures of walking and cognitive task performance under single-task and dual-task conditions. On average, 18 strides (27 steps) per person were analyzed for each single-task and dual-task walking condition. During single-task walking, people with PD had slower gait speeds than HYA (post hoc: *t*(28); *P* < .001; *d* = 2.38) and showed a trend toward slower speeds than HOA (post hoc: *t*(28); *P* = .024; *d* = .96), with large effect sizes for both comparisons. Single-task stride length was shorter for people with PD compared to HYA (post hoc: *t*(28); *P* < .001; *d* = 2.05). There were no between-group differences in single-task cadence, step width, or any measure of stability. For the cognitive task, people with PD had longer single-task response latencies and had lower response accuracy than HYA (post hoc: *t*(28); *P* < .001; *d* = 2.27 and *t*(28); *P* = .003; *d* = 1.45, resp.).

**Figure 1:**
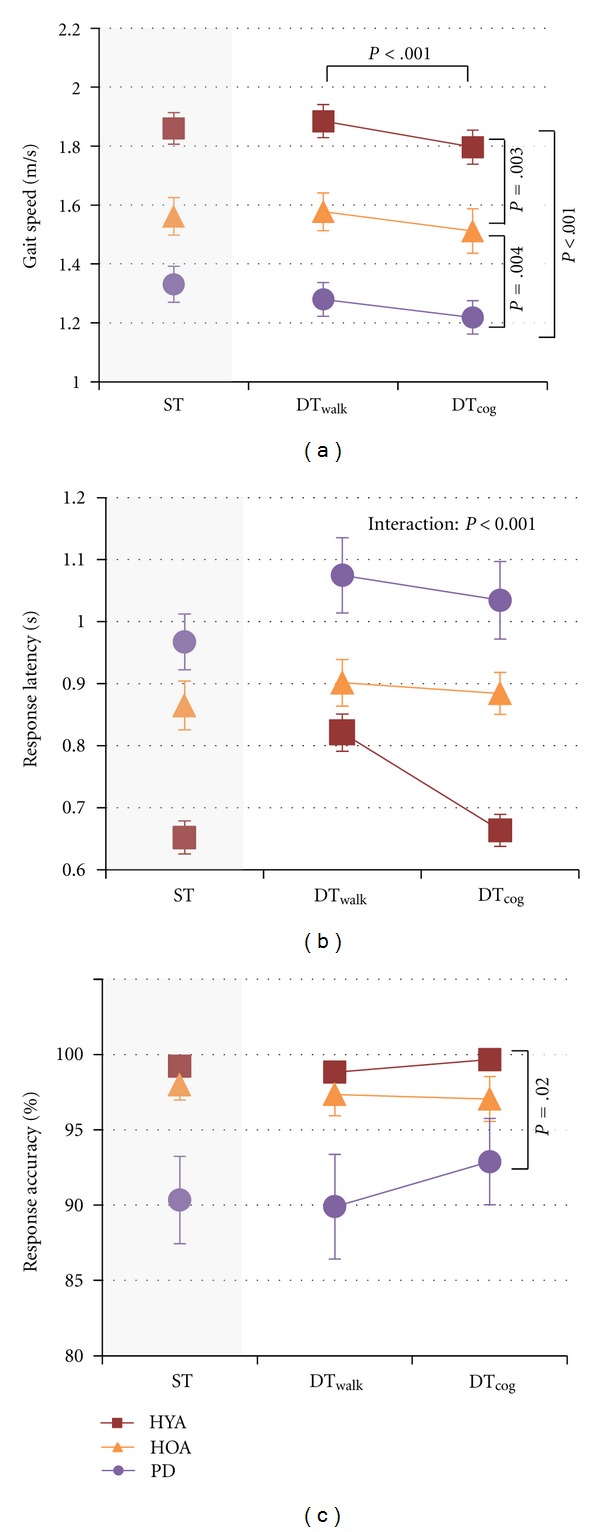
Gait speed (a), response latency (b), and response accuracy (c) for all groups in the single-task (ST; for reference) and both dual-task conditions. Red squares show data for healthy young adults. Orange triangles show data for healthy older adults. Purple circles show data for people with PD. Symbols represent means and bars show standard errors.

Under dual-task conditions, gait speed differed between groups (*F*(2,42) = 23.91; *P* < .001; *η*_*p*_^2^ = .53; Figure 1(a)). People with PD were slower than HYA (post hoc: *t*(28); *P* < .001; *d* = 2.68) and HOA (post hoc: *t*(28); *P* = .004; *d* = 1.21), and HOA were slower than HYA (post hoc: *t*(28); *P* = .003; *d* = 1.21). Effect sizes were large for both comparisons. Instructions had a large and significant effect on gait speed, with faster speeds in the DT_walk_ compared to the DT_cog_ condition for all groups (*F*(1,42) = 27.25; *P* < .001; *η*_*p*_^2^ = .39). There was no interaction between group and instructions for gait speed.

Stride length (*F*(2,42) = 18.91; *P* < .001; *η*_*p*_^2^ = .47) and cadence (*F*(2,42) = 4.07; *P* = .02; *η*_*p*_^2^ = .16) also differed between groups. Stride length was shorter for people with PD (post hoc: *t*(28); *P* < .001; *d* = 2.40) and HOA (post hoc: *t*(28); *P* = .001; *d* = 1.52) compared to HYA, but similar between people with PD and HOA (post hoc: *t*(28); *P* = .12; *d* = 0.69). Although people with PD had lower cadence than HYA or HOA, pairwise comparisons were not significant with *post hoc* analysis. Compared to the DT_cog_ condition, walking in the DT_walk_ was characterized by longer stride length (*F*(1,42) = 34.98; *P* < .001; *η*_*p*_^2^ = .45) and higher cadence (*F*(1,42) = 13.60; *P* = .001; *η*_*p*_^2^ = .25). Step width was not different between groups and was not affected by instructions. There was no interaction between group and instructions for any of the secondary spatiotemporal measures.

Measures of stability did not show a consistent pattern. There was a significant interaction between group and instructions for step width variability (*F*(2,42) = 3.69; *P* = .03; *η*_*p*_^2^ = .15), but no main effects of group or instructions. In DT_cog_ compared to DT_walk_, HYA decreased variability while HOA and people with PD increased variability. There was a trend toward group differences in stride time variability (*F*(2,42) = .2.71; *P* = .08; *η*_*p*_^2^ = .11), with lower variability in HYA compared to people with PD. Stride time variability was not influenced by instructions, and there was no interaction. For the frontal plane inclination angle, there were no effects of group or instruction and no interaction.

 Dual-task cognitive performance differed between groups. There was a significant interaction between group and instructed focus for response latency (*F*(2,42) = 9.20; *P* < .001; *η*_*p*_^2^ = .31; Figure 1(b)). Response latency was longer for people with PD than HYA and longer in the DT_walk_ than the DT_cog_ condition, but the effect of instructions on response latency was significant only for HYA (post hoc: *t*(14); *P* < .001; *d* = 1.49). Response accuracy differed between groups (*F*(2,42) = 4.55; *P* = .02; *η*_*p*_^2^ = .18; Figure 1(c)), with lower response accuracy in people with PD compared to HYA (post hoc: *t*(28); *P* = .016; *d* = 1.12).

### 3.3. Dual-Task Performance: Relative Measures

**Figure 2:**
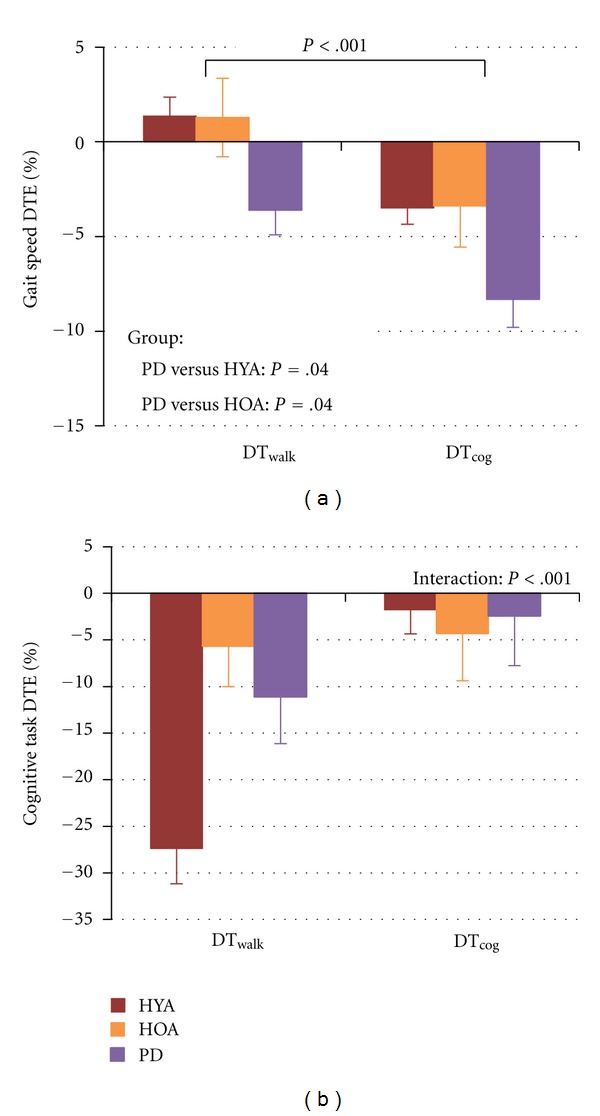
The effects of instructions on walking DTEs (a) and composite cognitive task DTEs (b) for the healthy young adults (red bars), healthy older adults (orange bars), and people with PD (purple bars).

Table 3 and Figure 2 show relative measures of dual-task performance. Walking DTEs differed between groups (*F*(2,42) = 4.40; *P* = .02; *η*_*p*_^2^ = .17). People with PD had greater dual-task costs than HOA (post hoc: *t*(28); *P* = .04; *d* = .69) and HYA (post hoc: *t*(28); *P* = .04; *d* = .97), though differences were not significant after correction for multiple comparisons. Walking dual-task costs were smaller in the DT_walk_ compared to DT_cog_ condition (*F*(1,42) = 27.62; *P* < .001; *η*_*p*_^2^ = .40). There was no interaction between group and instructions for walking DTEs.

There was an interaction between instructions and group for response latency DTEs (*F*(2,42) = 14.92; *P* < .001; *η*_*p*_^2^ = .42). The effect of instructions on response latency dual-task costs was greatest for the HYA (post hoc: *t*(14); *P* < .001; *d* = 1.50), with larger dual-task costs in the DT_walk_ compared to the DT_cog_ condition. There was a trend toward greater dual-task costs for response accuracy in the DT_walk_ compared to the DT_cog_ condition (*F*(1,42) = 2.94; *P* = .09; *η*_*p*_^2^ = .065). There was an interaction between instructions and group for the composite cognitive task DTEs (*F*(2,42) = 11.73; *P* < .001; *η*_*p*_^2^ = .36; Figure 2(b)). Composite cognitive dual-task costs were greater in the DT_walk_ compared to the DT_cog_ condition. This effect was significant and moderate for people with PD (post hoc:* t*(14); *P* = .015; *d* = .69) and was significant and large for HYA (post hoc: *t*(14); *P* < .001; *d* = 1.51).

### 3.4. Relative Magnitude of Dual-Task Performance Changes

For walking, all groups demonstrated similar magnitudes of performance changes in response to instructions, as assessed by walking mAAI values (Table 3). Groups differed with respect to the cognitive task mAAI (*F*(2,42) = 11.68; *P* < .001; *η*_*p*_^2^ = .36). Both people with PD (post hoc: *t*(28); *P* = .008; *d* = 1.12) and HOA (post hoc:* t*(28); *P* < .001; *d* = 1.68) had smaller relative magnitude of cognitive task performance changes compared to HYA, with large effect sizes for both comparisons.

## 4. Discussion

This study examined the effects of instructions on dual-task performance in people with PD compared to healthy adults. Instructions influenced both walking and cognitive task performance among people with PD. People with PD, like HOA and HYA, walked faster and had smaller gait speed dual-task costs when instructed to focus on walking. However, when focused on walking, both people with PD and HYA demonstrated declines in composite cognitive task performance. The magnitude of dual-task walking changes in response to instructions was comparable for all groups. In contrast, the relative magnitude of cognitive task performance changes was similar for people with PD and HOA, but reduced compared to HYA.

As hypothesized, people with PD, like HYA and HOA, were able to modify dual-task performance in response to instructions. For people with PD, instructions had a large effect on gait speed and gait speed dual-task costs and a moderate effect on composite cognitive dual-task costs. These findings are consistent with previous research demonstrating that instructions affect gait speed in healthy young and older adults [19, 20, 26] and in people with PD [13, 31], suggesting that dual-task performance and prioritization are dynamic.

For people with PD, instructions to focus on walking reduced gait speed dual-task costs but resulted in modest increases in the composite cognitive dual-task cost. This finding provided some support for the hypothesis that improvements in walking would be associated with declines in cognitive task performance among people with PD. In previous research, instructions improved walking but had mixed effects on concurrent task performance in people with PD. In one study, no declines in a concurrent motor task were observed [13], while another study demonstrated a trend towards declines in a concurrent cognitive task [31]. Differences in the nature and difficulty of tasks and the ability to quantify concurrent tasks may contribute to these differing findings. In addition, we asked participants to walk as quickly as they safely could, which may have increased the cognitive demands over those associated with self-paced walking, thus contributing to cognitive task declines in the DT_walk_ condition.

Finally, we hypothesized that the relative magnitude of performance changes in response to instructions would be smaller in people with PD compared to healthy adults. The current study provides mixed support for this hypothesis. Instructions affected walking to a similar degree in all groups despite slower gait speeds and greater dual-task costs in the PD group. However, both people with PD and HOA demonstrated a reduced ability to modify cognitive task performance compared to HYA. This suggests that instructions remain an effective way to modify dual-task walking in people with PD and HOA, but the ability to modify concurrent cognitive task performance may be limited in these groups relative to young adults. Previous research has shown smaller effects of instructions in people with PD [32] and in older adults [19, 32] compared to young adults. Though not statistically significant, the authors suggest that these differences may reflect reduced cognitive flexibility among older adults and people with PD.

Inappropriate prioritization of concurrent tasks over walking has been proposed to contribute to dual-task walking decrements in people with PD [17, 33]. Our results are not consistent with a fixed and invariant “posture-second” strategy in PD. Instead, they demonstrate that dual-task performance can be modified by instructions, suggesting that prioritization is dynamic. However, this interpretation should be made cautiously as the inference of prioritization from dual-task performance is challenging [32]. Direct comparisons of walking and concurrent task performance are limited because the relative scale and sensitivity of outcome measures may differ. In addition to prioritization, performance may be influenced by a number of other factors, like fatigue. Further research is needed to clarify mechanisms underlying dual-task walking deficits in PD and to determine the constellation of individual, task, and environmental factors that influence dual-task performance in people with PD [16].

Within each task, certain parameters were more affected by instructions than others. Instructions affected gait speed for all groups, but measures of stability were not consistently affected. The number of strides or steps used to calculate variability measures may have been low relative to some recommendations [34] but were consistent with previous research [35] and recent recommendations specific to people with PD [36]. The fact that instructions did not affect measures of stability suggests that improved speed did not come at the expense of maintaining stability in this sample. For the cognitive task, the effect of instructions on response latency and accuracy varied across groups. Instructions affected only response latency in HYA. Neither parameter was affected by instructions in HOA, but instructions affected both latency and accuracy in people with PD. Poorer accuracy among the group with PD may have been a reflection of mild cognitive changes as indicated by MoCA scores. Future studies should consider the effect of instructions on multiple aspects of dual-task performance, as the observed effects may vary based on factors such as the specific tasks performed and differences in individual characteristics (e.g., cognitive function) across populations.

Several limitations of this paper should be noted. First, the use of instructions to walk as fast as possible may have provided some implicit focus on walking for all groups. Fast-as-possible walking was chosen to maximize the difficulty of the tasks and the potential for interference. However, if fast-as-possible walking resulted in a persistent focus on walking, it may have led to an underestimate of dual-task interference, particularly in people with PD who may use a cognitive strategy to modify walking. Secondly, the composite cognitive task DTE measure was calculated as the sum of response latency and response accuracy DTEs. This calculation reflects equal weighting or importance of these two parameters and may oversimplify the relationship between speed and accuracy in the cognitive task. This measure was used to account for any within-task trade-offs (e.g., improvement in response latency but decline in response accuracy) and variable performance changes among different participants (e.g., declines in latency alone for one individual but declines in accuracy alone for another participant).

There are several important clinical implications of this research. First, people with PD retain the ability to modify dual-task performance in response to instructions. Improvements in gait speed did not come at the expense of stability, suggesting that the use a cognitive strategy (i.e., to focus on walking) is a viable rehabilitation approach to improve walking under dual-task as well as single-task conditions in people with PD. However, under dual-task conditions, the use of a cognitive strategy to focus on walking may be associated with a decline in concurrent task performance, thus limiting the utility of this approach in functional settings. In particular, certain situations, such as crossing a busy street or navigating a crowded environment, may require fast and accurate cognitive processing in order to avoid a collision or fall. Under such situations, any decline in cognitive processing resulting from a focus on walking may pose a threat to safety. Whether or not this trade-off between walking and cognitive task performance can be ameliorated with continued practice is unknown.

## 5. Conclusions

This research demonstrates that dual-task performance can be modified by instructions in people with PD, but walking improvements may come at a cost to cognitive task performance. The ability to modify walking in response to instructions appears to be preserved in people with PD compared to healthy individuals, even though dual-task walking deficits are greater in people with PD compared to healthy young and older adults. The ability to walk while performing concurrent tasks and the ability to modify dual-task performance to meet the demands of different tasks and environments are critical to mobility in daily life. Future research should continue to examine factors that influence dual-task performance among people with PD.

## Figures and Tables

**Table 1:** Individual characteristics and group summary for participants with PD and summary for HOA and HYA groups.

	Age	PD duration	Sex	MDS-UPDRS	H & Y	MoCA	Medications (LED)
PD-101	66	13	M	32	2	29	Carbidopa/levodopa, pramipexole (256)
PD-102	67	7	F	21	2	26	Carbidopa/levodopa, pramipexole, selegiline (204)
PD-103	77	3	M	41	2	27	Carbidopa/levodopa (63)
PD-104	62	17	F	47	2	26	Carbidopa/levodopa, entacapone, amantadine, ropinirole (958)
PD-105	72	6	F	18	3	27	Carbidopa/levodopa, rasagiline (163)
PD-106	66	4	F	9	1	29	Rasagiline, pramipexole (125)
PD-107	78	9	M	31	2	22	Carbidopa/levodopa, pramipexole (525)
PD-108	81	2	M	46	2	27	Carbidopa/levodopa (75)
PD-109	70	7	M	57	2	30	Carbidopa/levodopa, pramipexole (450)
PD-110	70	8	F	18	2	25	Carbidopa/levodopa (200)
PD-111	83	4	M	22	2	29	Carbidopa/levodopa (150)
PD-112	79	6	M	49	3	23	Carbidopa/levodopa, amantadine (425)
PD-113	71	4	M	38	2	28	Ropinirole (160)
PD-114	68	1	M	25	2	29	Selegiline (100)
PD-115	73	1	F	23	3	29	Carbidopa/levodopa (125)

PD mean (SD) range	72.2 (6.2) 62–83	6.1 (4.4)	6 F/9 M	32 (14) 9–57	2.1 (1.5) 1–3	27.1 (2.3) 22–30	265 (238)

HOA mean (SD) range	69.2 (7.1) 60–80	—	10 F/5 M	—	—	28.5 (1.7) 24–30	—

HYA mean (SD) range	26.4 (4.3) 20–36	—	9 F/6 M	—	—	—	—

Age and PD duration in years. MDS-UPDRS: Movement Disorders Society Unified Parkinson's Disease Rating Scale, motor examination. H & Y: Hoehn and Yahr rating scale. MoCA: Montreal Cognitive Assessment. LED: levodopa equivalent dosage. SD: standard deviation.

**Table 2:** Mean (SD) values for walking and the cognitive task values in all groups under single-task (ST) and both dual-task conditions.

	ST	DT_walk_	DT_cog_
Gait speed (m/s)			
HYA	1.86 (.21)	1.88 (.22)	1.80 (.23)
HOA	1.56 (.25)	1.58 (.25)	1.51 (.29)
PD	1.33 (.24)	1.28 (.22)	1.22 (.22)

Stride length (m)			
HYA	1.68 (0.15)	1.68 (.15)	1.63 (.15)
HOA	1.43 (0.20)	1.40 (.21)	1.38 (.22)
PD	1.31 (0.20)	1.27 (.19)	1.24 (.20)

Cadence (steps/min)			
HYA	134 (15)	135 (16)	132 (16)
HOA	131 (12)	135 (13)	132 (15)
PD	122 (15)	122 (16)	119 (14)

Step width (m)			
HYA	0.14 (.02)	0.14 (.02)	0.14 (.02)
HOA	0.13 (.02)	0.14 (.03)	0.13 (.03)
PD	0.13 (.03)	0.13 (.04)	0.13 (.04)

Step width variability (%)			
HYA	15.1 (5.2)	15.7 (6.0)	13.3 (4.5)
HOA	17.8 (4.9)	16.2 (3.8)	17.9 (4.4)
PD	16.2 (5.0)	14.8 (5.0)	16.3 (4.5)

Stride time variability (%)			
HYA	2.3 (.7)	2.1 (.7)	2.0 (.5)
HOA	2.6 (1.2)	2.5 (.8)	2.3 (1.0)
PD	2.7 (1.0)	2.7 (1.0)	2.8 (1.4)

Inclination angle (degrees)			
HYA	7.4 (.6)	7.4 (.7)	7.5 (.7)
HOA	7.6 (1.0)	7.6 (1.0)	7.5 (1.1)
PD	7.5 (.9)	7.6 (1.0)	7.5 (.9)

Response latency (s)			
HYA	0.65 (.10)	0.82 (.12)	.66 (.10)
HOA	0.87 (.15)	0.90 (.15)	0.89 (.13)
PD	0.97 (.17)	1.08 (.24)	1.03 (.24)

Response accuracy (%)			
HYA	99.2 (1.1)	98.8 (2.3)	99.7 (.9)
HOA	98.0 (3.9)	97.4 (5.5)	97.1 (5.7)
PD	90.3 (11.2)	89.9 (13.5)	92.9 (11.1)

**Table 3:** Mean (SD) values for dual-task effects (DTE) and modified attention allocation index (mAAI) for walking and the cognitive task measures in all groups.

	HYA	HOA	PD
Gait speed DTE (%)			
DT_walk_	1.3 (3.9)	1.3 (8.0)	−3.6 (5.0)
DT_cog_	−3.5 (3.3)	−3.4 (8.4)	−8.3 (5.7)

Response latency (%)			
DT_walk_	−27.0 (14.6)	−5.1 (13.1)	−11.0 (14.1)
DT_cog_	−2.2 (9.6)	−3.5 (14.4)	−6.4 (10.0)

Response accuracy (%)			
DT_walk_	−0.4 (2.1)	−0.6 (5.1)	−0.1 (12.9)
DT_cog_	0.5 (1.1)	−0.9 (5.9)	4.0 (16.0)

Composite cognitive task (%)			
DT_walk_	−27.4 (14.6)	−5.7 (16.6)	−11.1 (19.4)
DT_cog_	−1.8 (10.0)	−4.3 (19.6)	−2.4 (20.8)

Walking mAAI (%)			
	4.8 (4.1)	4.7 (7.6)	4.7 (5.9)

Composite cognitive task mAAI (%)			
	25.6 (17.3)	1.4 (11.6)	8.7 (12.8)
